# Association between the school environment and children’s body mass index in Terengganu: A cross sectional study

**DOI:** 10.1371/journal.pone.0232000

**Published:** 2020-04-24

**Authors:** Sharifah Wajihah Wafa, Rasyidah Ghazalli

**Affiliations:** Faculty of Health Sciences, Universiti Sultan Zainal Abidin, Kuala Nerus, Terengganu, Malaysia; University of Idaho, UNITED STATES

## Abstract

With the on-going interest in implementing school policies to address the problem of childhood obesity in Malaysia, there is urgent need for information about the association between school environment and children’s weight status. This study aims to investigate the association between school environmental factors (physical, economic, political and sociocultural) with BMI of school children in Terengganu. The school environment factors were assessed using a set of validated whole-school environmental mapping questionnaires, consisting of 76 criteria with four domains; physical environment (41 criteria), economic environment (nine criteria), political environment (nine criteria) and sociocultural environment (17 criteria). This involved face-to-face interview sessions with 32 teachers from 16 schools (eight rural and eight urban). In addition, 400 school children aged between 9 and 11 years of the selected schools were assessed for BMI (WHO 2007 reference chart), dietary intake (food frequency questionnaire (FFQ)) and physical activity level (physical activity questionnaire for children (PAQ-C)). Multiple regression was used to examine the association between school environment factors and BMI of the school children. Seven school environment criteria were found to be associated with BMI of school children when it was adjusted for calorie intake and physical activity level. About 33.4% of the variation in BMI of school children was explained by health professional involvement, simple exercise before class, encouragement to walk/ride bicycle to/from school, no high-calorie food sold, healthy options of foods and drinks at tuck shop, availability of policy on physical activity and training teacher as a role model. Policy makers should make urgent actions to address the obesogenic features of school environments. It should strive towards setting up healthy school environment and improving school curricula to promote healthy behaviours among the school children.

## Introduction

The prevalence of childhood obesity in Malaysia has remained consistently high. Within 30 years, Malaysia has undergone a transition from under-nutrition to relative over-nutrition. The incidence of underweight children had decreased from 55% to 14.4% within a decade [1]. Meanwhile, findings from the National Health Morbidity Survey (2006) showed that the number of children aged 10 to 12 years classified as overweight had increased from 5.4% to 16.3% over the last decade [2, 3]. The survey also reported that primary school children (33.7%) had a higher prevalence of over nutrition (overweight and obesity) than secondary school adolescents (28.5%). This drastic escalation of obesity placed Malaysia as number one in Southeast Asia and number six in Asia in terms of obesity prevalence [4].

The epidemic of obesity in Malaysia increased along with socioeconomic and lifestyle transitions, which are commonly attributed to a combination of globalisation and urbanisation. Changes in dietary habits and sedentary lifestyles have been associated with the increasing prevalence of obesity irrespective of age, ethnicity and social status. Furthermore, the escalation of nutrition-related chronic degenerative diseases, once an urban phenomenon, has now spread to the rural population at an alarming rate. A lot of research has been devoted towards investigating possible factors that could contribute to childhood obesity, but research on school environment factors and its association with obesity is limited. The school environment is one of the concerns that influence children’s health-related behaviour; it has been regarded as the optimum setting to establish healthy eating behaviours and lifestyles among children [5–7]. Apart from being the most suitable place to target childhood obesity, schools also play a role in the development of children’s dietary practices and involvement in physical activity [8].

The Ministry of Health (MoH) recognises the school as the most significant avenue in managing obesity and promoting a healthy lifestyle among Malaysian school children [9]. The guide on the Management of Healthy School Canteen outlines the type of foods allowed to be sold, foods that are not recommended, and foods that are not allowed to be sold in school canteens; this guide has been implemented in schools since 2012. However, the compliance to the guideline has not been encouraging. Unhealthy foods are still widely available in school canteens. Research findings have suggested that improvements to the school food environment may enable students to make healthier food choices and lower their body mass index (BMI) [10]. Several studies have reported on the influence of the school environment on children’s health-related behaviours. A study conducted in China reported an association between the BMI of school children with the availability of soft drinks at school shops, the availability and the number of western food outlets in the school vicinity, as well as school curricula such as sports-meeting and health education session [11]. In the United States, school children who were offered French fries, similar potato products and desserts in subsidised school meals more than once weekly were associated with a significantly higher likelihood of obesity [12].

Understanding the potential factors in an environmental context that can impact children’s eating habits and physical activities would be the most effective strategy to address the problem of childhood obesity [13]. Combining environmental interventions with educational interventions enhances the impact on achieving healthy weight and helps to maintain this effect over the longer term. Furthermore, as policies are increasingly being used to improve the school environment, there is a need to gain a clearer understanding of the role played by the school environment on student’s BMI. In Malaysia, the evidence is limited with regards to the influence of school environment factors on health-related behaviours and the emerging trend of childhood obesity. Moreover, the evidence provided by the aforementioned studies may not be generalisable to developing countries such as Malaysia. Not only that, most previous studies underscore the importance of other factors such as economic environment, political environment and sociocultural environment in shaping the dietary and physical activity behaviours of school children and subsequently their weight status. Thus, it is imperative to investigate the school environment factors affecting children’s weight status. It is hoped that the findings from this study would help guide policymakers and decision makers to determine the best practices in childhood obesity prevention.

## Materials and methods

### Study design and study population

This study utilized a cross-sectional study design and was conducted in government primary schools in Terengganu, Malaysia. The sampling frame for this study was obtained from the Department of Education [14] website. There were 366 government primary schools located in Terengganu distributed across eight districts of Besut, Setiu, Hulu Terengganu, Kuala Nerus, Kuala Terengganu, Marang, Dungun and Kemaman. By using a computer-generated random number, eight of schools were selected. The population of school children aged between 9 and 11 in the 366 government primary schools in Terengganu was 39, 893. The sample size to represent the population was estimated to be 380 and was obtained using the formula introduced by Krejcie and Morgan (1970) as follows:

$$\begin{array}{ll} n & =\;\left[\left(3.841\right)\;\left(39\;893\right)\;\left(0.50\right)\;\left(1-0.50\right)\right]\;/\;\left\{\left[0.052\;\left(39\;893\;-1\right)\right]\;+\;\left[\left(3.841\right)\;\left(0.5\right)\;\left(1-0.50\right)\right]\right\}\\ & =\;38\;307.3\;/\;\left(99.73\;+\;0.96025\right)\\ & =\;380\;+\;5\%\;drop-out\;rate\\ & =\;400\;school\;children \end{array}$$

Finally, a stratified random sampling with non-proportional allocation was used to select the school children participating in this study. A total of 25 school children were recruited based on simple random sampling from each of 16 selected schools. The children were excluded if they had serious diseases such as diabetes mellitus, hypertension and cardiovascular disease. Meanwhile, the whole-school environmental mapping questionnaires were filled by each school’s teachers who were responsible for the school children’s affairs and the school curriculum according to the list of environmental factors in this study. Approval was obtained from the Ministry of Education [KP(BPPDP)603/5/JLD.02 (112)], Terengganu State Education Department (JPNT) [P.T. 06030-26(80)] and UniSZA Human Research Ethics Committee (UHREC) [UniSZA.C/1/UHREC/628-1(6)]. Permission to conduct the study was obtained from the principals of the respective schools. Finally, written parental consent and child assent were obtained prior to study commencement.

### Outcome measurements

Anthropometric measurements were obtained from all participants by trained researchers using standard methods. Body weight was measured using a digital weighing scale (Seca Robusta 813) to the nearest 0.1 kg, while height was measured using a portable stadiometer (Seca 213) to the nearest 0.1 cm. BMI was calculated using the WHO AntrhoPlus version 1.0.4 software. In this software, the cut-off for normal BMI-for-age is set between -2SD and +1 SD; the children are regarded as thin if the BMI-for-age is less than -2SD, deemed overweight when the BMI-for-age is between +1SD and +2SD, and regarded as obese if the BMI-for-age is more than +2SD [15].

The dietary intakes of the school children were assessed using a self-administered food frequency questionnaire (FFQ) consisting of 97 food and beverages (F&B) consumed by Malays, Chinese and Indians in Malaysia. The F&B items were subdivided into ten categories; whole-grain, meat, fish, eggs, vegetables, fruits, nuts, dairy products, local deserts (*kuih-muih*) and beverages. The questionnaire was adapted from the MyBreakfast Study [16]. Each participant was required to record the serving size of the food/beverage consumed by them for each meal. The intake frequency was based on their habitual intake from the last two months. The macro- and micro-nutrient intakes were then calculated using the Nutritionist Pro Inc. diet analysis software (Axxya System) and compared to the recommended nutrient intake for Malaysians [17].

The physical activity levels of the participants were recorded using a validated questionnaire adapted from the Physical Activity Questionnaire for Children (PAQ-C) instrument by Kowalski et al. [18] and the Child and Adolescent Physical Activity and Nutrition Survey (CAPANS-PA) recall questionnaire by Strugnell et al. [19]. The questionnaire is a Malay language version that has been validated in a previous study [2] to make it easier for the school children to answer the questions. It included the frequency of activities done by them in the previous week and the types of physical activities. The data were analysed based on PAQ-C scoring. The scores for questions number one to nine were between one and five. The physical activity level was determined using the mean scale of the nine items [20]. PAQ-C scores were then assigned as low activity (≤2), moderate activity (>2 and ≤3), and high activity (>3).

Variables on the school environment were obtained from a face-to-face interview with each school teacher using a whole-school environmental mapping questionnaire in Malay [13], which is based on the Analysis Grid for Environments Linked to Obesity (ANGELO) framework by Swinburn et al. [21] and ‘School Food Pack’ [22]. The questionnaire consisted of four domains of school environment factors; physical (what is available) with 41 items; economy (the costs involved) with nine items; political (the rules) with nine items; and sociocultural (attitudes and beliefs) with 17 items. Each item was addressed using an initial closed question (yes = agree with the statement, no = disagree with the statement), followed by an open question when the criteria were not met or further information regarding the items were required.

## Statistical analysis

Data analysis was conducted using the Statistical Package for Social Science (SPSS) version 24.0. The descriptive statistics were presented as means with standard deviation or percentage of prevalence. It was used to describe the characteristics of the participants in terms of BMI, dietary intake and physical activity levels.

Next, univariable and multivariable analyses were performed to determine the school environmental factors that were associated with the primary school children’s BMI, while controlling for calorie intake and physical activity level as covariates. In the first stage, simple linear regression was applied to determine the potential variable that was of great value for BMI. Variables with a p-value of or less than 0.25 were included for further multivariable analysis. The variables were included based on statistical significance as well as principles of parsimony and biological plausibility. Interaction, multicollinearity, model fitness and assumptions, outliers, as well as influential cases were checked. The final model was presented with the adjusted regression coefficients (b), 95% confidence intervals (CI), p-values, and coefficient of determinations (R^2^).

## Results

A total of 400 primary school children with a mean age of 10.45 (0.60) were involved in this study, comprising of 44.8% boys and 55.3% girls. A majority of them were of Malay ethnicity (99.5%). On average, the mean BMI was 20.74 (4.98) kg/m^2^ and the standardised mean BMI (z-score) was 1.26 (3.05). The prevalence of overweight and obesity was found to be 23.3% and 26.5%, respectively. For dietary assessment, the mean calorie intake of the participants was 1965 (290) kcal, consisting of 276.46 grams of carbohydrate, 71.15 grams of protein and 63.90 grams of fat. As depicted in Table 1, the mean physical activity score was 2.31 (0.45).

**Table 1:** Descriptive statistics of school children (n = 400).

Variables	Total n (%)	Mean (SD)
**Gender**		
Male	179 (44.8)	
Female	221 (55.3)	
**Age**		10.45 (0.60)
**Ethnicity**		
Malay	398 (99.5)	
Others	2 (0.5)	
**BMI z-score**		1.26 (0.35)
**BMI (kg/m**^**2**^**)**		20.74 (4.98)
Underweight	16 (4.0)	
Normal	185 (46.3)	
Overweight	93 (23.3)	
Obese	106 (26.5)	
**Dietary intake**		
Calorie (kcal)		1965.00 (290.00)
Carbohydrate (g)		276.46 (50.34)
Protein (g)		71.15 (11.13)
Fat (g)		63.90 (13.26)
**Physical Activity (CPAQ score)**		2.31 (0.45)
Low (<2)	112 (28.0)	
Moderate (>2 and ≤3)	270 (67.5)	
High (>3)	18 (4.5)	

Table 2 shows the factors associated with BMI. Kilocalorie (p<0.001) and physical activity (p = 0.059) were factors significantly associated with BMI, without controlling any confounding variables. Apart from that, 48 out of 76 school environment factors with p>0.25 in univariate analysis predicted the BMI of school children. For physical environment, BMI was negatively associated with teaching health and nutrition in the curriculum, health professional involvement (doctor or nurse visits), programmes involving health professionals (e.g. nutritionists and dietitians), health education for healthy eating, displaying information about healthy eating along school corridors, simple exercises available before class, walking or riding bicycles to school, information along the corridor about healthy lifestyles, visits to sports centres, facilities at school such as gyms, indoor halls, availability of footpaths and leisure rooms specific for health promotion, not selling high calorie foods, not selling high-calorie drinks, displaying healthy eating information and equality of food choices sold.

**Table 2:** Variables of body mass index by simple linear regression for school environmental mapping (n = 400).

Variable	Crude b^b^ (95% CI)	p-value
**Kilocalorie (kcal)**	0.005 (0.003, 0.006)	<0.001
**Physical Activity**	-1.043 (-2.124, 0.039)	0.059
**School Environment**		
**Physical Environment**		
Health and nutrition are taught in the curriculum		
No	0	
Yes	-2.483(-4.493, -0.474)	0.016
Health professional involvement (Doctor or nurse visits)		
No	0	
Yes	-3.456(-4.535, -2.377)	<0.001
Program involving health professionals (e.g. Nutritionist & dietitian) Motivation/promoting healthy eating and physically active		
No	0	
Yes	-2.952(-3.969, -1.936)	<0.001
Health education for healthy eating (promotion, information and program conducted by school teachers)		
No	0	
Yes	-0.821(-1.830, 0.188)	0.110
Display information about healthy eating along school corridor (e.g. Food calories posters)		
No	0	
Yes	-1.330(-2.806, 0.146)	0.077
Health education for physical activity (promotion, information and program conducted by school teachers)		
No	0	
Yes	1.724(-0.294, 3.741)	0.094
Simple exercise (stretching/warm-up) available before class		
No	0	
Yes	-3.401(-4.482, -2.320)	<0.001
Walking/riding bicycles to school encouraged		
No	0	
Yes	-1.235(-2.216, -0.255)	0.014
Information along the corridor about a healthy lifestyle		
No	0	
Yes	-2.554(-3.501, -1.607)	<0.001
Visit to sports centre		
No	0	
Yes	-1.504(-2.978, -0.030)	0.046
Gym		
No	0	
Yes	-2.765(-4.771, -0.759)	0.007
Indoor hall (use for any programme at school, indoor game like badminton, etc.)		
No	0	
Yes	-1.134(-2.384, 0.117)	0.075
Availability of footpath		
No	0	
Yes	-0.958(-1.966, 0.050)	0.062
Leisure room specific for health promotion		
No	0	
Yes	-1.082(-2.064, -0.100)	0.031
Calm canteen		
No	0	
Yes	-3.858(-5.290, -2.426)	<0.001
No high calorie foods sold (nuggets, sausage, etc.)		
No	0	
Yes	-1.293(-2.297, -0.289)	0.012
No high-calorie drink sold (fizzy, etc.)		
No	0	
Yes	-3.028(-4.120, -1.936)	<0.001
Healthy eating information displayed		
No	0	
Yes	-4.207(-6.188, -2.225)	<0.001
Healthy food choices positioned attractively at the front of the serving counter		
No	0	
Yes	2.030(0.562, 3.498)	0.007
Equality of food choices sold		
No	0	
Yes	-1.042(-2.049, -0.035)	0.043
Other free drinking water (free milk scheme, etc.)		
No	0	
Yes	1.118(-0.360, 2.595)	0.138
Free vegetables to all pupils (Notes: free only for Supplementary Feeding Scheme to pupils from low income family)		
No	0	
Yes	1.481(0.504, 2.458)	0.003
**Economic Environment**		
Rules/policy to monitor food sold outside the school gates		
No	0	
Yes	0.723(-0.262, 1.709)	0.150
Nutritious food sold near school (e.g. fruit)		
No	0	
Yes	1.417(0.414, 2.419)	0.006
Specific rules/policy to monitor tuck shop at school		
No	0	
Yes	-2.483(-4.493, -0.474)	0.016
Existence of healthy foods and drinks at tuck shop		
No	0	
Yes	-1.446(-2.416, -0.476)	0.004
Existence of low-fat snacks at tuck shop		
No	0	
Yes	-0.911(-1.895, 0.072)	0.069
**Politic Environment**		
National nutrition guidelines and food policy use for school canteen guideline and others related to food		
No	0	
Yes	-1.906(-3.376, -0.437)	0.011
Implementation of the guidelines at the canteen		
No	0	
Yes	-2.830(-4.285, -1.374)	<0.001
Information to families to prepare healthy meals at home and lunch box		
No	0	
Yes	1.443(0.441, 2.445)	0.005
Existence of policies for staff to attend training programs		
No	0	
Yes	1.259(0.209, 2.309)	0.019
Availability of policy for physical activity (specific)		
No	0	
Yes	-1.792(-2.834, -0.750)	0.001
Other programs or policy if any, in schools (breakfast, lunch or snacks)		
No	0	
Yes	1.371(0.368, 2.374)	0.008
**Sociocultural Environment**		
Food not used as a reward		
No	0	
Yes	2.398(0.389, 4.408)	0.020
Leading by example (training teacher as a role model)		
No	0	
Yes	2.204(1.093, 3.315)	<0.001
Leading by example (training food handlers as role models)		
No	0	
Yes	-2.483(-4.493, -0.474)	0.016
Celebrities invited for promoting healthy lifestyle		
No	0	
Yes	-3.080(-4.170, -1.990)	<0.001
No	0	
Yes	-2.813(-3.753, -1.873)	<0.001
Collaboration with the department of health		
No	0	
Yes	-1.886(-2.927, -0.846)	<0.001
Collaboration with the department of education		
No	0	
Yes	-2.029(-2.996, -1.061)	<0.001
Collaboration with the others (e.g. counsellor, public health service)		
No	0	
Yes	-2.708(-3.731, -1.685)	<0.001
Activities involving public, family and community		
No	0	
Yes	-1.489(-2.491, -0.488)	0.004
Network with other schools to promote healthy eating and physical activity		
No	0	
Yes	-3.134(-4.351, -1.917)	<0.001
Incentives or rewards to children who behavioural improvement (i.e. eating healthier or doing more physical activity)		
No	0	
Yes	-0.953(-1.960, 0.055)	0.064
Assessment for décor and seating arrangement		
No	0	
Yes	-2.223(-3.186, -1.260)	<0.001
Articles about healthy lifestyle for the school newsletter/website		
No	0	
Yes	-3.216(-4.663, -1.769)	<0.001
Barrier to implement healthy eating and doing physical activity regularly		
No	0	
Yes	1.287(0.162, 2.411)	0.025

^a^Simple linear regression;

^b^Crude regression coefficient

Furthermore, with regards to economic and political environment, BMI was negatively associated with specific rules/policy to monitor the tuck shop at school, existence of healthy foods and drinks as well as low-fat snacks at the tuck shop, national nutrition guidelines and food policy use for school canteen guidelines and others related to food, implementation of the guidelines at the canteen and availability of policies for physical activity were identified to be factors significantly associated with BMI, when no other confounding variables were considered.

For socio-cultural environment factors, BMI was negatively associated with training food handlers as role models, celebrities invited for promoting healthy lifestyles, availability of growing food at school, collaboration in promoting healthy eating and physical activity with the department of health, the department of education and others (e.g. counsellor, public health service), activities involving the public, family and community, networking with other schools to promote healthy eating and physical activity, incentives or rewards to children who behaviourally improve, assessment for decor and seating arrangement as well as articles about healthy lifestyle for the school newsletter/website.

As shown in Table 3, in the final model, physical environment criteria such as health professional involvement (adjusted b = -3.06, 95% CI: -4.33, -1.80, p < 0.001), simple exercise before class (adjusted b = -3.75, 95% CI: -4.88, -2.62, p < 0.001), encouragement to walk or ride bicycles to/from school (adjusted b = 1.12, 95% CI: 0.10, 2.15, p = 0.032) and no high-calorie food sold (adjusted b = -2.99, 95% CI: -3.99, -2.01, p <0.001) were significantly associated with the school children’s BMI, when these factors were adjusted for calorie intake and physical activity level. The existence of healthy foods and drinks at tuck shop (adjusted b = -1.75, 95% CI: -2.75, -0.75, p = 0.001), which was the economic environment criterion, and the available physical activity policy (adjusted b = -2.74, 95% CI: -3.96, -1.52, p < 0.001), which was the political environment criterion, were also significantly associated with the children’s BMI when they were adjusted for calorie intake and physical activity level. For sociocultural environment factors, having a training teacher as the role model (adjusted b = 1.50, 95% CI: 0.33, 2.67, p = 0.012) was significantly associated with the school children’s BMI, when it was adjusted for calorie intake and physical activity level. Overall, the study results found that the variation in BMI of 33.4% among the school children were explained by this equation; BMI (kg/m2) = 26.40–3.06 (Health professional involvement) - 3.75 (Simple exercise available before class) + 1.12 (Encouragement to walk/ride bicycle to/from school) - 2.99 (No high-calorie food) - 1.75 (Healthy foods and drinks at tuck shop) - 2.74 (Physical activity policy) + 1.50 (Training teacher as the role model), R2 = 0.334.

**Table 3:** The environmental factors associated with the BMI of school children in Terengganu.

Variables	Simple Linear Regression^a^		Multiple Linear Regression^b^	
	Crude b^c^ (95% CI)	p-value	Adjusted b^d^ (95% CI)	p-value
Health professional involvement	-3.46 (-4.54, -2.38)	<0.001	-3.06 (-4.33, -1.80)	<0.001
Simple exercise available before class	-3.40 (-4.48, -2.32)	<0.001	-3.75 (-4.88, -2.62)	<0.001
Encouragement of walking/riding bicycles to school	-1.24 (-2.22, -0.26)	0.014	1.12 (0.10, 2.15)	0.032
No high-calorie foods sold	-1.29 (-2.30, -0.29)	0.012	-2.99 (-3.99, -2.01)	<0.001
Existence of healthy foods and drinks at tuck shop	-1.45 (-2.42, -0.48)	0.004	-1.75 (-2.75, -0.75)	0.001
Availability of physical activity policy	-1.79 (-2.83, -0.75)	0.001	-2.74 (-3.96, -1.52)	<0.001
Leading by example (training teacher as a role model)	2.20 (1.09, 3.32)	<0.001	1.50 (0.33, 2.67)	0.012

Stepwise multiple linear regression methods were applied and the model reasonably fits. Model assumptions are fulfilled. There is no interaction and multicollinearity between the independent variables. Coefficient of determination (R^2^) = 0.334.

^a^Simple linear regression;

^b^Multiple linear regression.

^c^Crude regression coefficient;

^d^Adjusted regression coefficient.

Regression equation:

$$Y=\beta_{0}-\beta_{1}(X_{1})+\beta_{2}(X_{2})+\beta_{3}(X_{3})-\beta_{4}(X_{4})-\beta_{5}(X_{5})-\beta_{6}(X_{6})+\beta_{7}(X_{7})$$

BMI (kg/m^2^) = 26.40 - 3.06 (Health professional involvement) - 3.75 (Simple exercise before class) + 1.12 (Encouragement to walk/ride bicycle to/from school) - 2.99 (No high-calorie food) - 1.75 (Healthy foods and drinks at tuck shop) - 2.74 (Physical activity policy) + 1.50 (Training teacher as the role model).

## Discussion

The results indicated that generally, the school environment (physical, economic, political and sociocultural environments) tended to have facilities that promote healthy choices thus influencing the BMI of school children. The findings revealed that 33.4% of the variation in BMI of school children was explained by school environment factors. There were significant associations found between child weight status with the involvement of health professionals in health, nutrition and physical activity programmes, availability of simple exercises (stretching/warm-up) before class, encouragement to walk or ride bicycles to school, no high-calorie food sold at the school canteen, availability of healthy foods and drinks at tuck shop, providing physical activity policy and training teachers as role models. In addition, with respect to individual variables, calorie intake and physical activity were also significantly related to BMI.

Several school physical environmental factors remained linked with BMI after adjusting for calorie intake and physical activity level. First, students were less likely to have high BMI if they attended a school that involved health professionals such as doctor or nurse visits in health, nutrition and physical activity programmes. This is consistent with the services provided by the Ministry of Health Malaysia which provides school health services to school children to ensure optimum health care through health education campaigns or health camps at school. According to CDC [23] and previous literature [24], school health services could be more effective if increased attention was given to working collaboratively on partnerships and coordinated by a multidisciplinary team. Second, children were less likely to be overweight if they attended a school that has simple exercises (stretching/warm up) available before class and prohibit the availability of high-calorie food. Evidence from a systematic review by Turner and colleagues indicated that a variety of food- and activity-related factors were associated with student weight status and were found to be statistically significant [25]. However, these findings represent only a small proportion of the number and variety of school-level factors investigated compared to the present study. Researchers found that the possibility of doing simple increased energy expenditure tasks and promoting physical activities in the classroom results in positive changes in health-related behaviours among school children [26, 27]. In addition, the results revealed that improvement in terms of food and beverages available at school can help school children to make healthier food choices, thus reducing their BMI. The food and beverages available in schools have a significant impact on the children’s diet and weight, as they have access to them regularly [28]. 35 to 47% of children’s and adolescents’ dietary intake starts from school [29]. The findings of this study paralleled those from prior researches confirming that schools which restrict the availability of junk foods are associated with a lower rate of students with increased BMI and a lower proportion of overweight or obese students, while the schools that allow junk foods to be sold at school are associated with increased BMI among the students [30–35].

Even though encouragement to walk or ride bicycles to school also remained linked with BMI, the direction of the association changed; children walking or riding bicycles to school had higher BMIs compared to their counterparts who do not walk or ride bicycles to schools, which is counterintuitive. We had expected the encouragement to walk/ride a bicycle to be associated with better weight status. This finding indicates that walking or riding bicycles did not protect the children from being physically inactive. Given the increased exposure to sedentary activities in school children [36], it could have contributed to the weight status of school children. Apart from insufficient physical activity, excessive sedentary behaviour has been implicated as potential cause of obesity among children [37].

Among the school economic environmental factors, it was observed that the availability of healthy foods and drinks at tuck shop was significantly associated with BMI. This analysis supports an earlier study which found that the absence of school shops and snack bars as well as limiting the availability of less healthful foods in school shops were associated with reduced intake of sugar-sweetened beverages and energy dense snacks [29, 38]. Prior research indicated that the availability of healthy and nutritious food at school is significantly associated with consumption levels among school children [39]. Consistent with existing research, children attending schools that provided healthy foods and drinks at the tuck shop tended to have lower BMIs than their counterparts who were attending schools that sold less healthful foods [40]. In the aforementioned study conducted among children, the presence of fruit tuck shops due to school policy had a positive impact as more school children ate fruit as a snack at school [39]. Thus, a healthy tuck shop at school can be one of the strategies to increase healthy food choices among school children. Nonetheless, the evidence linking the availability of healthful foods to child weight status has been inconsistent. Researchers have not found the availability of healthful foods to be associated with healthy dietary intake and higher risk of obesity [41].

Availability of policy for physical activity may promote increased participation in physical activities among school children. Our results suggested that children attending schools that establish policies for physical activity had lower risk for increased BMI than their counterparts who were attending schools that did not provide policies for physical activity. Some existing research has linked the provision of physical activity’s policy to increased physical activity. Evidence from previous studies have indicated that implementation of a physical activity policy is associated with significant reduction in BMI and improvement in fitness level among school children [42–44]. In 2011, the Malaysian Education Ministry launched the “One Student One Sport” policy with the main aim of having a healthy social school environment. However, the success of “One Student, One Sport” policy depends on the sports facilities and equipment at the schools, the duration of sports periods and the design of physical education curriculum in order to attract the interest of students and make it part of their life culture [45]. This suggests that the physical activity policy should be implemented or enforced as it could contribute to the physical activity levels of school children and thus improve the child weight status.

Training teachers as role models may promote better weight status among school children [46]. However, the results of this study suggest that children attending schools that had trained teachers as role models had higher BMI. One possible explanation is that the children do not regard school staff as their role models in shaping their behaviour [47]. Children only look up to their teachers in terms of educational capacity instead of other areas in life [48, 49]. Notably, the home environment, an equally important context in which healthy lifestyles are developed in children [50, 51], was not captured in the present analyses. Prior research has indicated that parents have the biggest influence and are the main role models in a child’s life [52]. Children are more likely to adopt the same eating habits as their parents. Therefore, children are likely to adopt the same lifestyle habits as their parents. Likewise, in school, teachers receive little or no training in nutrition or obesity prevention measures, lack knowledge on nutrition and eating disorders, and lack confidence in dealing with students who experience eating disorders [53].

The strengths of the present study include exploring the association between school environment and obesity among school children by using school mapping adapted from the ANGELO framework as well as individual determinants such as eating pattern and physical activity engagement among school children. However, the present work has certain limitations which should be noted. Firstly, the cross-sectional study could not evaluate the causal relationship between school environment factors and weight status. At best, this study found that associations exist between the examined variables and the outcome variable. Furthermore, since the present work focused on school environment and BMI, the other limitation is that the study did not report on other factors associated with BMI at household and community levels influencing individual behaviours. Thus, further research on a hierarchical regression model that studies the factors involving the community, school, household, parents and students should be conducted in the future. The hierarchical regression approach is significant to explore the associated risk factors in the presence of interrelated factors at different levels as well as contribute to robust and unbiased effect size estimation. The understanding of the two analyses needs to be further scrutinised.

## Conclusion

The emphasis on investment and collaboration are essential for the prevention of childhood obesity. Without urgent collective action, Malaysia’s childhood obesity problem will be difficult to control. If childhood obesity rates further increase, many health complications in younger children as well as adolescents will escalate. In fact, the situation is dire because many obese children continue to have increased weight during adolescence and the severity of obesity worsens until adulthood. Therefore, addressing the obesogenic elements present in the school environment can be an effective strategy for obesity prevention as the school environment exerts significant influence on children’s behaviour.

## Supporting information

S1 Appendix (DOCX)
