# Supplementary material for: Association between the school environment and children’s body mass index in Terengganu: A cross sectional study
Source: PLoS One. 2020 Apr 24;15(4):e0232000. doi: 10.1371/journal.pone.0232000 (PMC7182194; doi:10.1371/journal.pone.0232000)
Supplement: S1 Appendix — (DOCX) [file pone.0232000.s001.docx]

Associated Factors of Body Mass Index by Simple Linear Regression for Physical School Environmental Mapping (n = 400)

| **Criteria for physical environment** | **Regression Coefficient (ß)^a^** | **95% CI** | **p-value** |
| --- | --- | --- | --- |
| **Curriculum and education resources** |  |  |  |
| Health and nutrition are taught in the curriculum |  |  |  |
| No | 0 |  |  |
| Yes | -2.483 | -4.493, -0.474 | **0.016** |
| **Health, nutrition and physical activity programme** |  |  |  |
| Health professional involvement (Doctor or nurse visits) |  |  |  |
| No | 0 |  |  |
| Yes | -3.456 | -4.535, -2.377 | **<0.001** |
| Programme involving health professionals (e.g. Nutritionist & dietitian) Motivation/promoting healthy eating and physically active |  |  |  |
| No | 0 |  |  |
| Yes | -2.952 | -3.969, -1.936 | **<0.001** |
| Health education for healthy eating (promotion, information and programme conducted by school teachers) |  |  |  |
| No | 0 |  |  |
| Yes | -0.821 | -1.830, 0.188 | **0.110** |
| Display information about healthy eating along school corridor (e.g. Food calories posters, etc) |  |  |  |
| No | 0 |  |  |
| Yes | -1.330 | -2.806, 0.146 | **0.077** |
| Food calorie guidelines or other leaflets/books to students |  |  |  |
| No | 0 |  |  |
| Yes | 0.264 | -0.991, 1.519 | 0.679 |
| Health education for physical activity (promotion, information and programme conducted by school teachers) |  |  |  |
| No | 0 |  |  |
| Yes | 1.724 | -0.294, 3.741 | **0.094** |
| Annual sport event |  |  |  |
| No | 0 |  |  |
| Yes | -1.109 | -3.131, 0.912 | 0.281 |
| Simple exercise (stretching/warm-up) available before class |  |  |  |
| No | 0 |  |  |
| Yes | -3.401 | -4.482, -2.320 | **<0.001** |
| Walking/riding bicycle to school encouraged |  |  |  |
| No | 0 |  |  |
| Yes | -1.235 | -2.216, -0.255 | **0.014** |
| Provision physical activity guidelines or other leaflets/ books to children |  |  |  |
| No | 0 |  |  |
| Yes | 0.147 | -0.910, 1.204 | 0.785 |
| Information along the corridor about a healthy lifestyle |  |  |  |
| No | 0 |  |  |
| Yes | -2.554 | -3.501, -1.607 | **<0.001** |
| Visit to sports centre |  |  |  |
| No | 0 |  |  |
| Yes | -1.504 | -2.978, -0.030 | **0.046** |
| Visit to farm or food factory |  |  |  |
| No | 0 |  |  |
| Yes | -0.665 | -1.918,0.589 | 0.298 |
| **Facilities at school** |  |  |  |
| Gym |  |  |  |
| No | 0 |  |  |
| Yes | -2.765 | -4.771, -0.759 | **0.007** |
| Indoor hall (use for any programme at school, indoor game like badminton etc.) |  |  |  |
| No | 0 |  |  |
| Yes | -1.134 | -2.384, 0.117 | **0.075** |
| Availability of footpath |  |  |  |
| No | 0 |  |  |
| Yes | -0.958 | -1.966, 0.050 | **0.062** |
| Leisure room specific for health promotion |  |  |  |
| No | 0 |  |  |
| Yes | -1.082 | -2.064, -0.100 | **0.031** |
| **Break time and canteen environment** |  |  |  |
| Attractive canteen |  |  |  |
| No | 0 |  |  |
| Yes | -1.109 | -3.131, 0.912 | 0.281 |
| Calm canteen |  |  |  |
| No | 0 |  |  |
| Yes | -3.858 | -5.290, -2.426 | **<0.001** |
| **Menu provision** |  |  |  |
| No high calorie foods sold (nuggets, sausage etc.) |  |  |  |
| No | 0 |  |  |
| Yes | -1.293 | -2.297, -0.289 | **0.012** |
| No high-calorie drink sold (fizzy etc.) |  |  |  |
| No | 0 |  |  |
| Yes | -3.028 | -4.120, -1.936 | **<0.001** |
| No snack foods |  |  |  |
| No | 0 |  |  |
| Yes | -0.422 | -1.434, 0.589 | 0.412 |
| Healthy eating information displayed |  |  |  |
| No | 0 |  |  |
| Yes | -4.207 | -6.188, -2.225 | **<0.001** |
| Healthy food choices positioned attractively at the front of the serving counter |  |  |  |
| No | 0 |  |  |
| Yes | 2.030 | 0.562, 3.498 | **0.007** |
| Equality of food choices sold |  |  |  |
| No | 0 |  |  |
| Yes | -1.042 | -2.049, -0.035 | **0.043** |
| **Healthy food/drink subsidize** |  |  |  |
| Free drinking water (water cooler machines etc.) |  |  |  |
| No | 0 |  |  |
| Yes | 0.610 | -0.446, 1.665 | 0.257 |
| Other free drinking water (free milk scheme etc.) |  |  |  |
| No | 0 |  |  |
| Yes | 1.118 | -0.360, 2.595 | **0.138** |
| Free fruits to all pupils (Notes: free only for Supplementary Feeding Scheme to pupils from low income family) |  |  |  |
| No | 0 |  |  |
| Yes | -0.121 | -1.109, 0.867 | 0.810 |
| Free vegetables to all pupils (Notes: free only for Supplementary Feeding Scheme to pupils from low income family) |  |  |  |
| No | 0 |  |  |
| Yes | 1.481 | 0.504, 2.458 | **0.003** |
| Free food or drink during extracurricular activities in the evening to all pupils |  |  |  |
| No | 0 |  |  |
| Yes | 0.054 | -0.934, 1.042 | 0.915 |

* For model with dependent variable BMI value (kg/m^2^), the following variables: education resources (e.g. Food pyramid, food models, etc.), using educational resources (e.g. Food pyramid & food models, systematically), compulsory physical activity session among children at school, availability of sport centre, availability of playground (field), equipment/toys to encourage physical activity, break time available at schools for eating, pleasant and cheerful canteen, relaxing canteen, and clean canteen are constants. Statistics cannot be computed.

Associated Factors of Body Mass Index by Simple Linear Regression for Economic School Environmental Mapping (n = 400)

| **Criteria for economic environment** | **Regression Coefficient (ß)^a^** | **95% CI** | **p-value** |
| --- | --- | --- | --- |
| **Mobile caterer** |  |  |  |
| No mobile caterers near schools |  |  |  |
| No | 0 |  |  |
| Yes | -0.553 | -1.684, 0.577 | 0.336 |
| Rules/policy to monitor food sold outside the school gates |  |  |  |
| No | 0 |  |  |
| Yes | 0.723 | -0.262, 1.709 | **0.150** |
| Nutritious food sold near school (e.g. Fruit) |  |  |  |
| No | 0 |  |  |
| Yes | 1.417 | 0.414, 2.419 | **0.006** |
| **Tuck shop** |  |  |  |
| Specific rules/policy to monitor tuck shop at school |  |  |  |
| No | 0 |  |  |
| Yes | -2.483 | -4.493, -0.474 | **0.016** |
| Existence of healthy foods and drinks |  |  |  |
| No | 0 |  |  |
| Yes | -1.446 | -2.416, -0.476 | **0.004** |
| Existence of low-calorie versions of fizzy drinks, no added sugar fruit juices, low fat milk or water |  |  |  |
| No | 0 |  |  |
| Yes | -0.269 | -1.326, 0.788 | 0.617 |
| Existence of low-fat snacks |  |  |  |
| No | 0 |  |  |
| Yes | -0.911 | -1.895, 0.072 | **0.069** |
| Promotion leaflets for healthy eating/physical activity at tuck shop |  |  |  |
| No | 0 |  |  |
| Yes | -0.798 | -2.277, 0.682 | 0.290 |

* For model with dependent variable BMI value (kg/m^2^), the variable of tuck shop available at schools is constants. Statistics cannot be computed.

Associated Factors of Body Mass Index by Simple Linear Regression for Political School Environmental Mapping (n = 400)

| **Criteria for political environment** | **Regression Coefficient (ß)^a^** | **95% CI** | **p-value** |
| --- | --- | --- | --- |
| **National nutrition guidelines & food policy** |  |  |  |
| National nutrition guidelines and Food policy use for school canteen guideline and others related to food |  |  |  |
| No | 0 |  |  |
| Yes | -1.906 | -3.376, -0.437 | **0.011** |
| Implementation of the guidelines at the canteen |  |  |  |
| No | 0 |  |  |
| Yes | -2.830 | -4.285, -1.374 | **<0.001** |
| Rules for children to bring food to school |  |  |  |
| No | 0 |  |  |
| Yes | -0.151 | -1.131, 0.829 | 0.762 |
| Information to families to prepare healthy meals at home and lunch box |  |  |  |
| No | 0 |  |  |
| Yes | 1.443 | 0.441, 2.445 | **0.005** |
| **Policy for healthy education** |  |  |  |
| Existence of policies for staff to attend training programs |  |  |  |
| No | 0 |  |  |
| Yes | 1.259 | 0.209, 2.309 | **0.019** |
| **Policy for physical activity** |  |  |  |
| Availability of policy for physical activity (specific) |  |  |  |
| No | 0 |  |  |
| Yes | -1.792 | -2.834, -0.750 | **0.001** |
| **Responsibility for the policy** |  |  |  |
| Deciding the types of food to be provided (e.g. Catering etc) |  |  |  |
| No | 0 |  |  |
| Yes | -0.160 | -1.292, 0.972 | 0.781 |
| Other programs or policy if any, in schools (breakfast, lunch or snacks) |  |  |  |
| No | 0 |  |  |
| Yes | 1.371 | 0.368, 2.374 | **0.008** |

* For model with dependent variable BMI value (kg/m^2^), the variable of Monitoring/enforcement of the guideline at school is constants. Statistics cannot be computed.

Associated Factors of Body Mass Index by Simple Linear Regression for Social-cultural School Environmental Mapping (n = 400)

| **Criteria for socio-cultural environment** | **Regression Coefficient (ß)^a^** | **95% CI** | **p-value** |
| --- | --- | --- | --- |
| **Food beliefs, culture and preferences** |  |  |  |
| Trial/alternatives to change food beliefs, culture and preferences |  |  |  |
| No | 0 |  |  |
| Yes | -0.177 | -1.433, 1.078 | 0.782 |
| **Use of food as a reward** |  |  |  |
| Food not used as a reward |  |  |  |
| No | 0 |  |  |
| Yes | 2.398 | 0.389, 4.408 | **0.020** |
| **Leading by example-role models** |  |  |  |
| Leading by example (training teacher as a role model) |  |  |  |
| No | 0 |  |  |
| Yes | 2.204 | 1.093, 3.315 | **<0.001** |
| Leading by example (training food handlers as role models) |  |  |  |
| No | 0 |  |  |
| Yes | -2.483 | -4.493, -0.474 | **0.016** |
| Celebrities invited for promoting healthy lifestyle |  |  |  |
| No | 0 |  |  |
| Yes | -3.080 | -4.170, -1.990 | **<0.001** |
| **Growing food** |  |  |  |
| Growing food at school |  |  |  |
| No | 0 |  |  |
| Yes | -2.813 | -3.753, -1.873 | **<0.001** |
| **Collaboration in promoting healthy eating and physical activity** |  |  |  |
| Collaboration with the department of health |  |  |  |
| No | 0 |  |  |
| Yes | -1.886 | -2.927, -0.846 | **<0.001** |
| Collaboration with the department of education |  |  |  |
| No | 0 |  |  |
| Yes | -2.029 | -2.996, -1.061 | **<0.001** |
| Collaboration with the others (e.g. counselor, public health service etc.) |  |  |  |
| No | 0 |  |  |
| Yes | -2.708 | -3.731, -1.685 | **<0.001** |
| Collaboration with the private sector |  |  |  |
| No | 0 |  |  |
| Yes | 0.602 | -0.454, 1.657 | 0.263 |
| Activities involving public, family and community |  |  |  |
| No | 0 |  |  |
| Yes | -1.489 | -2.491, -0.488 | **0.004** |
| Network with other schools to promote healthy eating and physical activity |  |  |  |
| No | 0 |  |  |
| Yes | -3.134 | -4.351, -1.917 | **<0.001** |
| Committee/working group for school health promotion |  |  |  |
| No | 0 |  |  |
| Yes | 0.454 | -0.525, 1.433 | 0.363 |
| Incentives or rewards to children who behavioral improvement (i.e. eating healthier or doing more physical activity) |  |  |  |
| No | 0 |  |  |
| Yes | -0.953 | -1.960, 0.055 | **0.064** |
| Assessment for décor and seating arrangement |  |  |  |
| No | 0 |  |  |
| Yes | -2.223 | -3.186, -1.260 | **<0.001** |
| Articles about healthy lifestyle for the school newsletter/website |  |  |  |
| No | 0 |  |  |
| Yes | -3.216 | -4.663, -1.769 | **<0.001** |
| **Barrier in promoting healthy eating and physical activity in school** |  |  |  |
| Barrier to implement healthy eating and doing physical activity regularly |  |  |  |
| No | 0 |  |  |
| Yes | 1.287 | 0.162, 2.411 | **0.025** |
